# Pulmonary Artery Intimal Sarcoma Mimicking Intraluminal Seeding: A Case Report

**DOI:** 10.70352/scrj.cr.25-0504

**Published:** 2025-11-20

**Authors:** Akira Akazawa, Jun Hanaoka, Takuya Shiratori, Yo Kawaguchi

**Affiliations:** 1 Department of General Thoracic Surgery, National Hospital Organization Higashi-Ohmi General Medical Center, Higashi-Ohmi, Shiga, Japan; 2 Division of General Thoracic Surgery, Department of Surgery, Shiga University of Medical Science, Otsu, Shiga, Japan

**Keywords:** pulmonary artery sarcoma, intimal sarcoma, cardiopulmonary bypass, skip lesions, pneumonectomy

## Abstract

**INTRODUCTION:**

Pulmonary artery intimal sarcoma (PAIS) is a rare vascular malignancy. Complete surgical resection is crucial for favorable outcomes; however, intraoperative delineation of the tumor extent poses a significant challenge.

**CASE PRESENTATION:**

A 63-year-old woman with suspected PAIS underwent a left pneumonectomy under cardiopulmonary bypass. Despite macroscopically clear margins, histopathological examination of the pulmonary artery (PA) stump exhibited tumor infiltration, necessitating reevaluation. We identified multiple skip-like intraluminal nodules along the PA and performed resection and subsequent reconstruction utilizing an autologous pericardial patch. The final pathology demonstrated not only nodular lesions but also creeping intimal infiltration in segments without gross abnormalities.

**CONCLUSIONS:**

Although PAIS typically exhibits contiguous spread along the intima, the presence of skip-like nodules and subtle creeping infiltration is rare, complicating intraoperative assessment. Comprehensive intraoperative and pathological assessments are essential to achieve a complete resection.

## Abbreviations

FDG-PET
: fluorodeoxyglucose-PET
PA
: pulmonary artery
PAIS
: pulmonary artery intimal sarcoma
RVOT
: right ventricular outflow tract
SUVmax
: maximum standardized uptake value

## INTRODUCTION

PAIS is an aggressive neoplasm originating from multipotent mesenchymal stem cells within the intimal layer of the PA and is characterized by an undifferentiated morphology or heterologous differentiation, such as rhabdomyosarcoma and chondrosarcoma.^1)^ The incidence of PAIS is extremely low, estimated at 0.001%–0.03%. A systematic review and pooled analysis encompassing just over 600 reported cases has only recently been published.^1,2)^ The prognosis of untreated cases remains poor, with a survival of only ~1.5–3 months. Previous reports indicate that chemotherapy and radiotherapy provide limited benefit, whereas complete surgical resection may extend median survival to 36.5 ± 20.2 months. Conversely, incomplete resection has been associated with a median survival of 11 ± 3 months.^3)^ The attainment of complete resection depends on meticulous intraoperative evaluation of tumor extent and precise identification of surgical margins. PAIS generally advances by replacing the vascular endothelium, with intraoperative frozen section analysis serving to verify negative margins. However, in certain cases, the tumor manifests as multiple, discontinuous nodular lesions distributed along the vasculature, in a segmental or so-called “skip lesion” pattern, suggestive of intravascular metastasis. Here, we report a case of PAIS that demonstrated a progressive pattern, necessitating additional resection and vascular reconstruction.

## CASE PRESENTATION

**Fig. 1:**
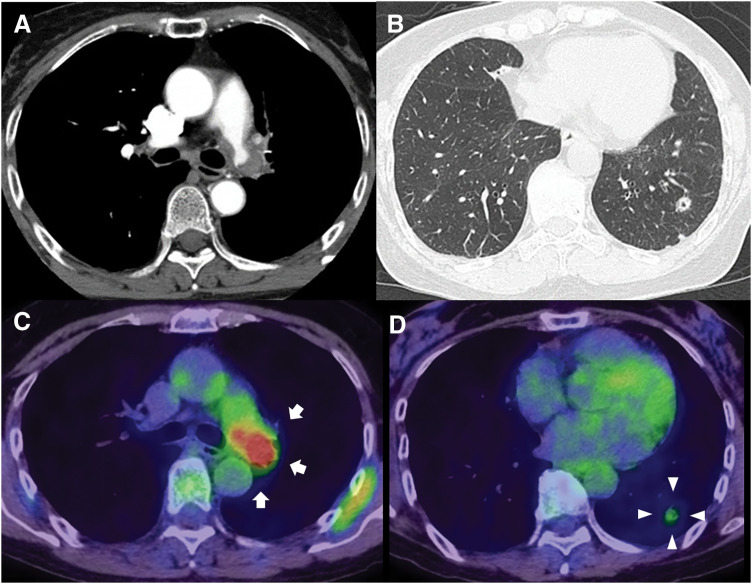
Preoperative imaging findings. Contrast-enhanced chest CT showing a mass encircling and compressing the left main pulmonary artery (**A**). Chest CT showing a cavitary mass in the left lower lobe using lung window settings (**B**). PET scan highlighting the left pulmonary artery (arrows) with a maximum standardized uptake value (SUVmax) of 13.3 (**C**), and a nodule (arrowheads) in the left lower lobe with an SUVmax of 2.0 (**D**).

A 63-year-old woman was referred to our hospital after a routine health checkup revealed chest abnormalities. She developed hemoptysis soon after a consultation at a different medical facility. Chest CT revealed thickening of the PA wall, resulting in luminal occlusion, and a cavitary nodule with spiculated margins in the left lower lobe (**Fig. 1A**, **1B**). FDG-PET demonstrated SUVmax of 13.3 in the thickened left PA wall and an SUVmax of 2.0 in the left lower lobe nodule (**Fig. 1C**, **1D**). Bronchoscopic transbronchial lung biopsy and CT-guided biopsy of the nodule revealed no evidence of malignancy. Moreover, laboratory tests, including blood D-dimer levels, revealed no coagulation abnormalities. Levels of all measured tumor markers associated with lung cancer were within the normal range. PAIS rather than pulmonary thromboembolism was suspected, with no evidence of distant metastasis. Therefore, surgical resection was performed to confirm a definitive diagnosis with curative intent. Given the inability to ascertain the distal extent of tumor invasion along the PA preoperatively, left pneumonectomy was considered the most appropriate procedure to ensure complete resection. Preoperative cardiac function was normal, although pulmonary function testing revealed a reduced diffusion capacity (%diffusing capacity of the lungs for carbon monoxide, 69.7%). Pulmonary perfusion scintigraphy confirmed a complete absence of perfusion to the left lung. Taken together, these findings indicate that left pneumonectomy is unlikely to result in a clinically significant reduction in pulmonary function; therefore, the procedure was considered both safe and appropriate. If the surgical margins could not be defined during dissection, tumor resection was planned under cardiopulmonary bypass with femoral arterial perfusion and venous drainage via the right atrium and femoral vein. Given the risk of embolization from tumor involvement around the PA bifurcation during central cannulation, femoral cannulation was performed instead.

**Fig. 2:**
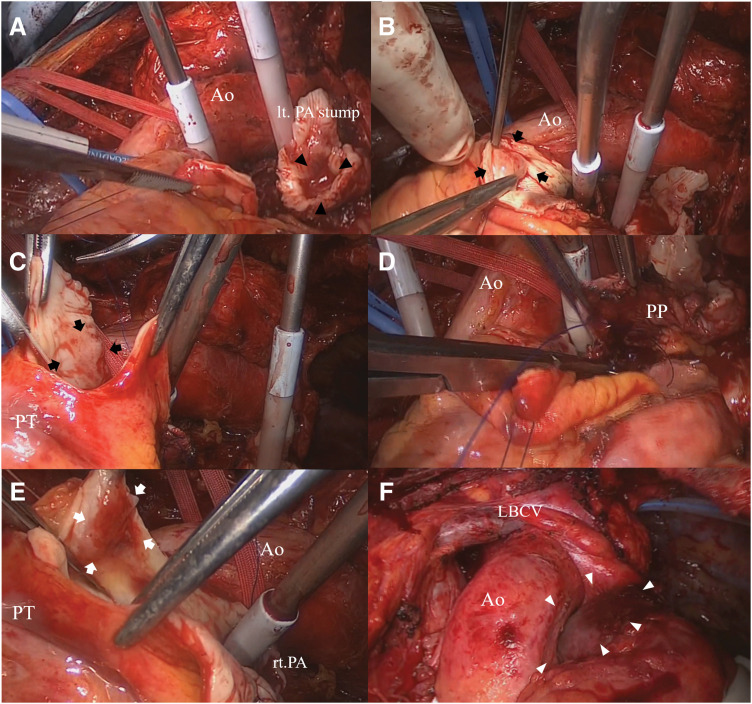
Intraoperative findings. Transection of the left pulmonary artery is performed with a safe margin from the primary tumor (black arrowheads) (**A**). A nodule with intimal thickening (black arrows) is identified at the bifurcation of the pulmonary trunk, located apart from the primary lesion; therefore, resection included this site (**B**, **C**). The arterial defect is reconstructed using an autologous pericardial patch and continuous sutures (**D**). Re-inspection revealed nodular tumor growth (white arrows) on the pulmonary trunk wall, located proximal to the primary tumor and extending toward the pulmonary valve (**E**). Final appearance after further resection and patch reconstruction (white arrowheads), displaying the completed repair (**F**). Ao, Aorta; LBCV, left brachiocephalic vein; lt, left; PA, pulmonary artery; PT, pulmonary trunk; PP, pericardial patch; rt, right

Surgery was performed via a sternotomy. Owing to palpable thickening extending proximally along the left PA, cardiopulmonary bypass was initiated. The left PA was transected at a location that ensured a safe margin from the primary tumor (**Fig. 2A**). Careful inspection of the arterial lumen revealed a nodule with intimal thickening at the bifurcation of the pulmonary trunk, positioned slightly apart from the primary tumor (**Fig. 2B**). The resection was subsequently extended to include this site (**Fig. 2C**). The defect was repaired using an autologous pericardial patch and a continuous 4-0 Prolene suture (**Fig. 2D**). Intraoperative frozen section analysis of the PA stump revealed residual tumor cells. Consequently, a portion of the sutured, reconstructed pericardial patch was excised, and additional resection was carried out. Reinspection of the intraluminal surface revealed nodular tumor growth along the wall of the pulmonary trunk, extending from a site slightly distal to the initial resection margin in the vicinity of the pulmonary valve (**Fig. 2E**). These areas were resected and reconstructed using another pericardial patch (**Fig. 2F**). An intraoperative frozen section examination after additional resection confirmed the absence of tumor cells. After terminating cardiopulmonary bypass, the left superior and inferior pulmonary veins were transected intrapericardially and extrapericardially. The left main bronchus, which exhibited no tumor invasion, was preserved, and the procedure was classified as a complete pneumonectomy. The total operative time was 504 min. Mechanical ventilation was discontinued on POD 3, and the patient was discharged without complications on POD 15.

**Fig. 3:**
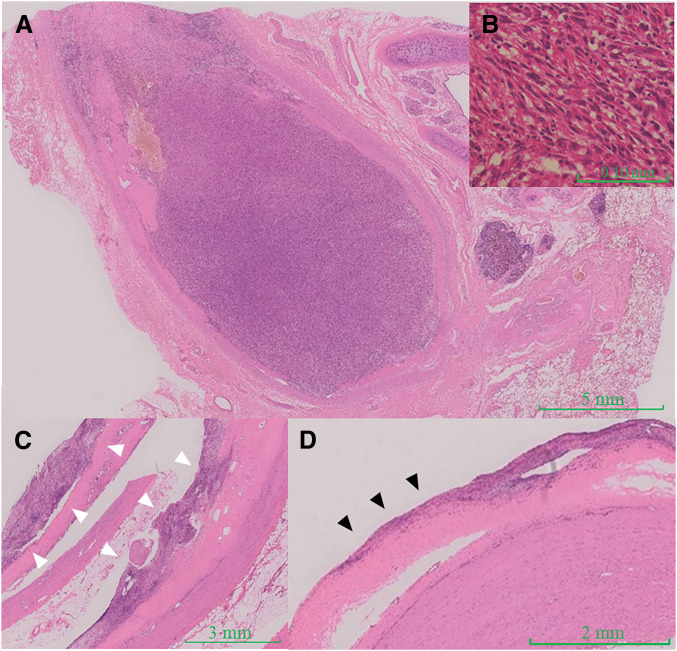
Postoperative pathological findings (hematoxylin and eosin staining). The tumor predominantly proliferated along the vascular intima, infiltrating the lumen of the left pulmonary artery (**A**). Histologically, the tumor exhibited diffuse and fascicular growth of spindle and polygonal cells, with nuclear atypia, pleomorphism, and infiltration of lymphocytes and plasma cells, consistent with undifferentiated pleomorphic sarcoma (**B**). Marked intimal thickening (white arrowheads) is observed in macroscopically abnormal vessels (**C**), while tumor cells also spread with intimal infiltration along the intima of vessels (black arrowheads) without gross changes (**D**).

Postoperative pathological examination with hematoxylin and eosin staining revealed that the tumor primarily proliferated along the vascular intima and infiltrated in a manner that occluded the lumen (**Fig. 3A**, **3B**). Immunohistochemical analysis of the resected specimen revealed focal positivity for CD34 and partial positivity for MDM2 in the tumor cells at the resection margin, whereas staining for CD31, AE1/AE3, SMA, desmin, and CDK4 was negative, indicating no specific line of differentiation. Based on these findings, a definitive diagnosis of an undifferentiated pleomorphic sarcoma was established. Marked intimal thickening due to tumor cell proliferation was observed in vessel segments with grossly apparent abnormalities (**Fig. 3C**). In contrast, tumor cells can spread along the intima in a creeping manner, even in vessels that appear macroscopically normal (**Fig. 3D**). The resected left lower lobe nodule demonstrated alveolar collapse with fibrosis and peribronchial lymphoid follicle formation with no evidence of malignancy.

The patient was subsequently followed up at the referral hospital without receiving adjuvant therapy. Two years and 9 months postoperatively, FDG-PET revealed abnormal uptake in the stomach. Upper gastrointestinal endoscopy revealed an elevated lesion in the gastric fundus, with the biopsy findings suggestive of metastatic recurrence. Total gastrectomy was performed, and histopathological examination revealed interlacing spindle cells with enlarged atypical nuclei within the lamina propria. Immunohistochemistry revealed AE1/AE3(–), desmin (–), myogenin(–), SMA(–), S100(–), CD31(–), CD34(–), and MDM2(+) expression, consistent with the findings from the pulmonary resection specimen, confirming metastatic recurrence. Simultaneously, the patient developed symptoms of right-sided heart failure, including dyspnea and leg edema. Right heart catheterization confirmed pulmonary hypertension, prompting the initiation of medical management. One year and 10 months following the onset of right heart failure, chest CT revealed an irregular hypodense lesion in the PA stump, representing a local recurrence. In consideration of the patient’s poor general condition and personal wishes, palliative care was provided. The patient died 5 years and 3 months after the surgery.

## DISCUSSION

Surgical intervention improves the prognosis of PAIS, while such improvement is limited to cases where complete resection is accomplished.^3)^ Although distant recurrences due to tumor embolism may occur, local recurrences are common.^4,5)^ Primary PAIS typically arises in the main PA or pulmonary valve region,^6,7)^ with the tumor extending distally along the intima in the direction of blood flow or proximally toward the RVOT.^5)^ RVOT invasion can be noted even during the early stages of disease.^8)^ Approximately 30% of cases involve the pulmonary valve, with some requiring valve replacement.

In this case, the tumor presented grossly as multiple, discontinuous nodules distributed along the vessel, exhibiting a segmental or “skip lesion” pattern indicative of intravascular metastasis. Histopathological examination revealed tumor cells infiltrating the intima, even in vessel segments, without apparent macroscopic abnormalities. Notably, in advanced cases of vascular sarcoma, the intimal thickness reportedly reaches 0.1–0.2 mm, compared with only 0.01–0.02 mm in normal PA intima.^9)^ However, even this degree of thickening is subtle and difficult to detect visually or by palpation, making intraoperative assessment of tumor extent extremely challenging. To our knowledge, only one previously reported case of PAIS has demonstrated a similar “skip lesion” pattern.^10)^ In this case, the primary tumor involved the right main PA, with a skip lesion in the left PA, for which surgical resection was undertaken. However, the resected specimen revealed positive margins, suggesting tumor progression along the intimal surface, as observed in our case.

Intraoperatively, resection was guided by grossly visible intimal thickening, with a negative surgical margin initially. Additional resection revealed several isolated intravascular nodules. These findings suggested that the extent of tumor spread may have been underestimated owing to the submission of limited intraoperative samples based on gross findings, resulting in the potential overlooking of microscopically infiltrative tumor components in areas that appeared unremarkable to the naked eye. This case highlights the need for caution during intraoperative pathological assessments by thoroughly examining the entire circumference of the vascular wall at the resection margins.

Although tumor localization is typically assessed preoperatively using CT and FDG-PET, no abnormalities were detected in this patient before surgery. Despite the existence of general recommendations for safe resection margins,^9)^ achieving an optimal balance between curability, surgical safety, and tolerance remains essential in clinical practice. This experience underscores the limitations of relying solely on imaging or gross inspection to determine the resection margins. Therefore, meticulous intravascular inspection, potentially aided by cardiopulmonary bypass, along with comprehensive intraoperative pathological evaluation, is necessary. For frozen section analysis, obtaining wide-ranging and strategically planned samples is important, regardless of the presence or absence of visible abnormalities.

This surgical procedure is highly invasive and can significantly impact postoperative hemodynamics, which may preclude some patients from receiving adjuvant therapy. Nonetheless, patients who received adjuvant chemotherapy and/or radiotherapy postoperatively exhibited improved survival rates compared with those who underwent surgery alone. Thus, the general postoperative condition should be carefully assessed before considering additional treatments.^11)^ Notably, while the present patient has remained recurrence-free for over 3 years, PAIS typically tends to recur relatively early after surgery. Careful surveillance is essential, particularly during the 1st postoperative year.

Recent molecular studies on intimal sarcomas, including PAIS, have demonstrated recurrent amplification of MDM2, MDM4, and CDK6, which appear to be largely mutually exclusive.^12)^ In addition, co-amplification of PDGFRA, CDK4, and TERT has been reported,^13)^ indicating alternative pathways for tumor progression. Although MDM2 amplification is observed in most cases, serving as a useful diagnostic marker,^14)^ PDGFRA amplification alone lacks specificity. Deoxyribonucleic acid methylation profiling has also emerged as a robust adjunct for distinguishing intimal sarcomas from histologically similar sarcomas, particularly in MDM2-negative cases.^12)^ Alterations in cell cycle regulators, such as p16, and oncogene activation, including c-MYC or KIT/PDGFRA, further suggest potential therapeutic implications.^15)^ These molecular insights not only facilitate accurate diagnosis but may also guide future targeted approaches in PAIS.

## CONCLUSIONS

In this case, we observed an unusual pattern of tumor progression, with intravascular dissemination and creeping infiltration along the intima. These findings emphasize that gross intimal thickening may not be a reliable indicator of tumor involvement. Careful intraoperative observation and strategic pathological examination are essential for an accurate diagnosis and complete resection.

## DECLARATIONS

### Availability of data and materials

The datasets supporting the conclusions of this article are included within the article.
